# Behavioral Variant Frontotemporal Degeneration: Exploring the Diagnostic Journey

**DOI:** 10.1002/alz70857_100942

**Published:** 2025-12-25

**Authors:** Carrie F Milliard, Shana G Dodge, Sweatha Reddy, Robert Reinecker, Mary Krause, Penny A Dacks

**Affiliations:** ^1^ FTD Disorders Registry, King of Prussia, PA, USA; ^2^ The Association for Frontotemporal Degeneration (AFTD), King of Prussia, PA, USA; ^3^ FTD Disorders Registry, LLC, King of Prussia, PA, USA; ^4^ The Association for Frontotemporal Degeneration, King of Prussia, PA, USA

## Abstract

**Background:**

Behavioral variant frontotemporal degeneration (bvFTD) is a form of FTD characterized by progressive changes in behavior, personality, and executive function. Key symptoms include apathy, disinhibition, loss of empathy, lack of insight, and speech and language difficulties. The overlap of these symptoms with psychiatric and neurological disorders frequently leads to misdiagnosis and substantial delays in obtaining an accurate diagnosis, causing frustration and distress for patients and their families.

**Method:**

The FTD Disorders Registry is a non‐profit, direct‐to‐participant registry relaunched in 2024 with a new platform, research, and electronic remote consent protocol. Between May 2024 and the data freeze on January 3, 2025, 205 participants in the United States and Canada completed a survey on the diagnostic journey, of whom 50 specified bvFTD as the current known diagnosis.

**Result:**

50 respondents described the journey to getting a bvFTD diagnosis, including persons diagnosed (*n* = 16), legally authorized representatives (*n* = 11), and care partners/reporters (*n* = 23). Respondents across roles noted the most distressing symptoms were mood changes (46%), problems with thinking and judgement (40%), memory issues (36%), and personality (36%) changes. The majority of respondents (76%) reported waiting more than a year from the onset of symptoms to receive a diagnosis, with 30% experiencing delays of 3 to 6 years and over half (66%) consulting 3 or more doctors before getting the correct diagnosis. Additionally, 52% of respondents were initially misdiagnosed, often receiving multiple other diagnoses including depression (73%), other psychiatric diagnoses (58%), anxiety (50%), and mild cognitive impairment (38%).

**Conclusion:**

Given the respondents’ demographics, these findings are likely conservative. While based on a limited sample size, these data align with findings from the FTD Insights survey of 1800 participants. They underscore the challenges and complexities involved in diagnosing bvFTD, the prevalence of misdiagnosis, and the significant impact it can have on patients' and caregivers’ lives. These findings highlight the need for targeted clinician education to recognize early signs of bvFTD and incorporate it into the differential diagnosis for patients with unexplained mood, cognitive, or personality changes. Future analyses will focus on larger sample sizes and utilizing validated tools to understand respondents’ socioeconomic and geographic factors correlate with diagnostic journey.

